# Educational exposure and sustainability-related outcomes among pharmacy students in Bulgaria: a cross-sectional study

**DOI:** 10.3389/fpubh.2026.1920936

**Published:** 2026-08-26

**Authors:** Evelina Gavazova, Hristo Manev, Daniela Kafalova

**Affiliations:** 1 Department of Organisation and Economics of Pharmacy, Faculty of Pharmacy, Medical University of Plovdiv, Plovdiv, Bulgaria; 2 Department of Medical Physics and Biophysics, Faculty of Pharmacy, Medical University of Plovdiv, Plovdiv, Bulgaria; 3 Research Institute, Medical University of Plovdiv, Plovdiv, Bulgaria

**Keywords:** cross-sectional survey, curriculum integration, environmental sustainability, healthcare sustainability, medicine disposal, pharmaceutical sustainability, pharmacy education, pharmacy students

## Abstract

**Introduction:**

Pharmaceutical sustainability is important for environmentally responsible health care, yet evidence regarding pharmacy students’ preparedness remains limited. This cross-sectional study assessed sustainability-related awareness, educational exposure, attitudes, knowledge of medication disposal, and behavior among pharmacy students in Bulgaria.

**Methods:**

An anonymous questionnaire was completed by 74 students from the Medical Universities of Plovdiv, Pleven, and Varna between April and December 2025. Descriptive statistics, chi-square tests, non-parametric tests, effect sizes, and exploratory binary logistic regression were applied.

**Results:**

Among 73 participants with valid data, 23 (31.5%) were familiar with sustainable pharmacy, 28 (38.4%) were partially familiar, and 22 (30.1%) were unfamiliar. Only 14 participants (18.9%) reported sustainability training, whereas 57 (77.0%) supported mandatory sustainability education. Thirty-two participants (43.2%) knew unused medicines could be handed over safely. Familiarity was associated with previous training (*p* < 0.001; Cramér’s V = 0.532) and year of study (*p* = 0.010; Cramér’s V = 0.371). Trained participants reported higher attitude scores (*p* = 0.030; r = 0.25). In the adjusted model, year of study was associated with safe-handover knowledge (*p* = 0.013), although estimates were imprecise.

**Conclusion:**

These findings identify gaps between positive attitudes, limited educational exposure, and knowledge. Structured sustainability education warrants further development and evaluation.

## 1 Introduction

Pharmaceutical sustainability extends beyond the management of pharmaceutical waste (1). It encompasses the environmental, social, and economic consequences of medicines throughout their life cycle, including research and development, manufacturing, procurement, distribution, prescribing, dispensing, use, and end-of-life management (2–4). It also includes reducing unnecessary medication use, supporting rational and environmentally informed prescribing, conserving healthcare resources, improving resilience in the pharmaceutical supply chain, and preventing active pharmaceutical ingredients from entering the environment (5). The global scale of pharmaceutical pollution was demonstrated in a study of 1,052 sampling sites across 104 countries, in which concentrations at 25.7% of sites exceeded levels considered safe for aquatic organisms or raised concerns regarding antimicrobial-resistance selection (6). Accordingly, sustainable pharmacy requires pharmacists to consider both patient outcomes and the broader environmental consequences of pharmaceutical decisions (7).

International evidence suggests that sustainability is not yet consistently embedded within pharmacy education. Australian pharmacy students have reported positive attitudes toward environmental sustainability but limited knowledge and curricular exposure (8). Studies from Spain and Canada have similarly identified gaps in students’ knowledge of pharmaceutical pollution and limited formal coverage of environmental sustainability within pharmacy curricula (9–11). A Swedish curriculum review found that environmental aspects of pharmaceuticals were included in some pharmacy courses, particularly regarding sustainable medicine use and pharmaceutical development, but were not consistently integrated across pharmacy programs (12). Educational interventions nevertheless suggest that sustainability concepts can be incorporated through case-based, interactive, and practice-oriented learning activities (13).

Evidence concerning sustainability education within Bulgarian pharmacy programs remains limited. Pharmacy education in Bulgaria is delivered through nationally regulated degree programs (14). Still, the extent to which pharmaceutical sustainability is explicitly and systematically addressed across institutions has not been established through a national curriculum audit. Examining students from several Bulgarian faculties can therefore provide an initial indication of their reported educational exposure, awareness, attitudes, and knowledge of medicine disposal. However, students’ self-reported exposure cannot establish whether particular sustainability competencies are formally present or absent from institutional curricula.

The study addressed the following research questions:
1. What levels of sustainable-pharmacy familiarity, educational exposure, attitudes, medicine-disposal knowledge, and self-reported disposal behavior are reported by pharmacy students in Bulgaria?
2. Are familiarity with sustainable pharmacy and attitudes toward pharmacists’ sustainability engagement associated with previous sustainability training or year of study?
3. Which educational and awareness-related factors are independently associated with students’ reported knowledge of local safe-handover arrangements for unused medicines?

## 2 Materials and methods

### 2.1 Study design and setting

A cross-sectional questionnaire-based study was conducted among undergraduate pharmacy students in Bulgaria. Responses were collected between 25 April and 3 June 2025. The study included students enrolled at the Faculties of Pharmacy of the Medical University of Plovdiv, the Medical University of Pleven, and the Medical University of Varna.

### 2.2 Participants, eligibility, and recruitment

Students were eligible to participate if they were enrolled in the first through fifth year of an undergraduate pharmacy degree program at one of the participating universities during the study period. Responses from individuals who did not provide informed consent or did not meet the eligibility criteria were excluded.

Participants were recruited through lecturers. The questionnaire was distributed as an anonymous online Google Forms survey. Participation was voluntary, and no financial or academic incentive was offered.

A total of 74 questionnaires were included in the analysis. Item-level denominators varied for two variables because one response was missing for familiarity with sustainable pharmacy and one was missing for awareness of Bulgarian sustainability regulation.

### 2.3 Questionnaire development

Data were collected using a self-administered questionnaire developed for this study. The literature informed the questionnaire. It was administered in Bulgarian and contained 10 substantive questions covering participants’ educational characteristics, awareness, prior educational exposure, attitudes, knowledge of medicine disposal, and self-reported disposal behavior.

Two pharmacy professors reviewed the initial questionnaire to assess the relevance, clarity, and comprehensibility of the items. Their feedback resulted in wording changes.

The questionnaire was pilot-tested among 5 pharmacy students who were not included in the final analytical sample. Participants in the pilot were asked to comment on item clarity, interpretation, survey length, and technical functionality. Based on the pilot, changes in wording were made.

Because the questionnaire primarily comprised individual categorical items and one single-item attitude rating rather than a multi-item psychometric scale, internal-consistency statistics such as Cronbach’s alpha were not considered applicable. The questionnaire assessed self-reported awareness, knowledge, attitudes, and behavior and was not designed as an objective or comprehensive measure of sustainability competence.

### 2.4 Variables and operational definitions

The following variables were included in the analysis:

Year of study: the participant’s current year in the pharmacy program, categorized from first to fifth year.

University: the Faculty of Pharmacy at which the participant was enrolled: Medical University of Plovdiv, Medical University of Pleven, or Medical University of Varna.

Familiarity with sustainable pharmacy: assessed using the question “Are you familiar with the term sustainable pharmacy?” Responses were categorized as “yes,” “partially familiar,” or “no”.

Previous educational exposure: defined as self-reported participation in a course or seminar dedicated to sustainable pharmacy and categorized as “yes” or “no”.

Awareness of Bulgarian regulation: assessed by asking whether Bulgaria had a law, ordinance, or other regulatory document establishing rules relating to sustainability in pharmacy. Responses were categorized as “yes,” “no,” or “do not know.” This variable represented perceived regulatory awareness rather than verified legal knowledge.

Knowledge of local safe-handover arrangements: defined as a “yes” response to the question asking whether the participant knew where and how unused medicines could be handed over for safe disposal in their city. This variable was distinguished from the broader question about knowing where to dispose of unwanted, expired, or damaged medicinal products.

General disposal knowledge: assessed by asking whether participants knew where to dispose of unwanted, expired, or damaged-package medicinal products. Responses were categorized as “yes” or “no”.

Self-reported disposal behavior: assessed by asking whether the participant had ever disposed of medicines in a sink or toilet. Responses were categorized as “yes,” “no,” or “do not remember”.

Support for mandatory sustainability education: assessed by asking whether sustainable-pharmacy education should be a compulsory component of the pharmacy curriculum. Responses were categorized as “yes,” “no,” or “unable to decide”.

Attitude toward pharmacists’ sustainability engagement: measured using a five-point rating ranging from 1 (“not at all important”) to 5 (“extremely important”). Higher scores indicated a greater perceived importance of pharmacists’ engagement in sustainable practices.

### 2.5 Statistical analysis

Statistical analyses were conducted using IBM SPSS Statistics. Categorical variables are presented as frequencies and percentages. Percentages were calculated using the number of valid responses for each item, and missing responses were reported separately. The attitude score is presented using the mean and standard deviation, together with the median and interquartile range.

Differences in attitude scores between students with and without prior sustainability training were evaluated using the Mann–Whitney U test. Differences across the five years of study were evaluated using the Kruskal–Wallis test. The effect size for the Mann–Whitney comparison was reported as r.

Associations between categorical variables were assessed using Pearson’s chi-square test. Because several contingency tables contained expected cell frequencies below five, exact or Monte Carlo *p*-values were also calculated. The magnitude of categorical associations was reported using Cramér’s V.

An exploratory multivariable binary logistic-regression model was fitted to examine factors associated with knowledge of local safe-handover arrangements for unused medicines. The dependent variable was coded as 1 for participants who reported knowing where and how unused medicines could be handed over for safe disposal in their city and 0 for those who did not. Predictors were selected *a priori* based on their conceptual relevance to educational exposure and sustainability awareness. They included familiarity with sustainable pharmacy, awareness of Bulgarian sustainability regulation, previous sustainability training, and year of study.

The reference categories were no familiarity with sustainable pharmacy, no awareness of Bulgarian sustainability regulation, no previous sustainability training, and first year of study. Adjusted odds ratios with 95% confidence intervals and *p*-values were reported. The regression was based on complete cases; 72 participants had complete information for the outcome and all predictors.

Overall model significance was evaluated using the likelihood-ratio chi-square test. Model calibration was assessed using the Hosmer–Lemeshow test, and explanatory performance was summarized using the Cox–Snell and Nagelkerke pseudo-R^2^ statistics. Classification accuracy was also calculated. Given the limited sample size, sparse categories, and the number of estimated parameters, the model was considered exploratory, and its estimates—particularly those with wide confidence intervals—were interpreted with caution.

All statistical tests were two-sided. A *p*-value below 0.05 was considered statistically significant. Statistical significance was interpreted together with effect sizes and confidence intervals.

### 2.6 Ethical considerations

Participation was voluntary and anonymous. Before accessing the questionnaire, participants received information about the purpose of the study, the voluntary nature of participation, the anonymous handling of responses, and their right to discontinue before submitting the questionnaire. Informed consent was obtained electronically before participation. No directly identifying personal information was collected.

Formal ethical review was not sought. The research team determined that ethical approval was not required because the study involved an anonymous, voluntary, non-interventional survey, collected no directly identifying information, and presented no more than minimal risk. This determination was made by the research team rather than formally confirmed by an institutional ethics committee. The study was conducted in accordance with applicable institutional and national requirements.

Categorical variables are reported as frequencies and percentages. Percentages were calculated using the number of valid responses for each item, and missing responses are reported separately.

## 3 Results

### 3.1 Participant characteristics and descriptive findings

A total of 74 pharmacy students were included in the study. Participants represented all five years of study: 20 (27.0%) were first-year students, 21 (28.4%) were second-year students, 18 (24.3%) were third-year students, 8 (10.8%) were fourth-year students, and 7 (9.5%) were fifth-year students. Participants were enrolled at the Medical University of Plovdiv, 44 (59.5%); the Medical University of Pleven, 19 (25.7%); and the Medical University of Varna, 11 (14.9%) (Table 1).

**Table 1:** Participant characteristics and descriptive results.

Characteristic or outcome	Category	*n* (%)
Year of study	First	20 (27.0)
	Second	21 (28.4)
	Third	18 (24.3)
	Fourth	8 (10.8)
	Fifth	7 (9.5)
University	Medical University of Plovdiv	44 (59.5)
	Medical University of Pleven	19 (25.7)
	Medical University of Varna	11 (14.9)
Familiarity with sustainable pharmacy*	Familiar	23 (31.5)
	Partially familiar	28 (38.4)
	Not familiar	22 (30.1)
Participation in sustainability training	Yes	14 (18.9)
	No	60 (81.1)
Awareness of a Bulgarian regulatory document*	Yes	23 (31.5)
	No	5 (6.8)
	Do not know	45 (61.6)
Knowledge of local safe-handover arrangements	Yes	32 (43.2)
	No	42 (56.8)
Knowledge of where to dispose of unwanted or expired medicines	Yes	43 (58.1)
	No	31 (41.9)
Disposed of medicines in a sink or toilet	Yes	9 (12.2)
	No	59 (79.7)
	Do not remember	6 (8.1)
Sustainability education should be mandatory.	Yes	57 (77.0)
	No	2 (2.7)
	Unable to decide	15 (20.3)
Importance of pharmacists’ engagement	Rating 5	38 (51.4)
	Rating 4	22 (29.7)
	Rating 3	14 (18.9)

*For familiarity and regulatory awareness, *n* = 73 because one response was missing. Percentages are based on valid responses. All other variables have *n* = 74.

Among the 73 participants who answered the question about familiarity with sustainable pharmacy, 23 (31.5%) reported being familiar with the term, 28 (38.4%) were partially familiar, and 22 (30.1%) were not familiar. One participant did not answer this question. Fourteen participants (18.9%) reported having previously participated in a course or seminar dedicated to sustainable pharmacy, whereas 60 (81.1%) reported no such educational exposure. Regulatory awareness was also limited. Among 73 valid responses, 23 participants (31.5%) believed that Bulgaria had a law, ordinance, or other regulatory document addressing sustainability in pharmacy, 5 (6.8%) believed that no such document existed, and 45 (61.6%) selected “do not know.” One response was missing.

The questionnaire included two separate indicators of medicine-disposal knowledge. Thirty-two participants (43.2%) reported knowing where and how unused medicines could be handed over for safe disposal in their city, whereas 42 (56.8%) did not. In response to the broader question concerning the disposal of unwanted, expired, or damaged-package medicinal products, 43 participants (58.1%) reported knowing where to dispose of such products, whereas 31 (41.9%) did not.

Nine participants (12.2%) reported having disposed of medicines in a sink or toilet, 59 (79.7%) reported that they had not done so, and 6 (8.1%) could not remember. Most participants, 57 (77.0%), believed that sustainable-pharmacy education should be a mandatory component of the pharmacy curriculum. Fifteen (20.3%) were unable to decide, and 2 (2.7%) disagreed.

Attitudes toward pharmacists’ involvement in sustainability were generally positive. Thirty-eight participants (51.4%) rated such involvement as extremely important, corresponding to a score of 5; 22 (29.7%) selected a score of 4; and 14 (18.9%) selected a score of 3. No participant selected a score of 1 or 2. The overall mean attitude score was 4.32 (SD = 0.78), and the median was 5.00 (IQR = 1.00) (Table 1).

### 3.2 Associations involving awareness, training, and year of study

Familiarity with sustainable pharmacy was associated with previous participation in sustainability-related training, χ^2^(2) = 20.680, *p* < 0.001, Cramér’s V = 0.532. Among participants familiar with sustainable pharmacy, 11 of 23 (47.8%) reported previous training. In comparison, training was reported by 1 of 28 (3.6%) partially familiar participants and 1 of 22 (4.5%) participants who were not familiar with the term. This analysis included 73 participants because one familiarity response was missing (Table 2).

**Table 2:** Associations of sustainable-pharmacy familiarity with educational exposure and year of study, and of safe-handover knowledge with medicine-disposal behavior.

Familiarity	No training	Training	Total
Not familiar	21	1	22
Familiar	12	11	23
Partially familiar	27	1	28
Valid total	60	13	73

Familiarity with sustainable pharmacy also differed by year of study, χ^2^(8) = 20.123, *p* = 0.010, Cramér’s V = 0.371. Familiarity was reported by 3 of 19 (15.8%) first-year students with valid responses, 4 of 21 (19.0%) second-year students, 5 of 18 (27.8%) third-year students, 6 of 8 (75.0%) fourth-year students, and 5 of 7 (71.4%) fifth-year students. These results indicate an association between study year and reported familiarity but do not establish that progression through the curriculum caused the observed differences.

No association was identified between knowledge of local safe-handover arrangements and self-reported disposal of medicines in a sink or toilet, χ^2^(2) = 0.262, *p* = 0.877, Cramér’s V = 0.059. Disposal in a sink or toilet was reported by 5 of 42 (11.9%) participants without local safe-handover knowledge and 4 of 32 (12.5%) participants with such knowledge (Table 2).

Several expected cell counts in these analyses were below five. Consequently, the results, particularly those involving smaller study-year and response categories, should be interpreted cautiously.

### 3.3 Attitudes toward pharmacists’ engagement in sustainability

Attitude scores did not differ significantly across years of study, Kruskal–Wallis H(4) = 2.564, *p* = 0.633. Median scores ranged from 4.00 to 5.00, and mean scores ranged from 4.11 among third-year students to 4.63 among fourth-year students (Table 3).

**Table 3:** Perceived importance of pharmacists’ engagement in sustainable practices by year of study and previous sustainability training.

Group	*n*	Mean (SD)	Median (IQR)	Test statistic	*p*-value	Effect size
Overall	74	4.32 (0.78)	5.00 (1.00)	—	—	—
Year of study				H = 2.564	0.633	—
First	20	4.35 (0.75)	4.50 (1.00)			
Second	21	4.38 (0.74)	5.00 (1.00)			
Third	18	4.11 (0.90)	4.00 (2.00)			
Fourth	8	4.63 (0.74)	5.00 (0.25)			
Fifth	7	4.29 (0.76)	4.00 (1.00)			
Previous sustainability training				U = 276.0	0.030	r = 0.25
Yes	14	4.71 (0.61)	5.00 (0.00)			
No	60	4.23 (0.79)	4.00 (1.00)			

Scores ranged from 1, “not at all important,” to 5, “extremely important.” Differences by year were evaluated using the Kruskal–Wallis test. Differences by training participation were evaluated using the Mann–Whitney U test. IQR, interquartile range; SD, standard deviation.

Participants who reported previous sustainability training had higher attitude scores than those without such training. The trained group had a mean score of 4.71 (SD = 0.61) and a median of 5.00 (IQR = 0.00), compared with a mean of 4.23 (SD = 0.79) and a median of 4.00 (IQR = 1.00) among participants without previous training. The difference was statistically significant, Mann–Whitney U = 276.0, *p* = 0.030, with a small-to-moderate effect size, r = 0.25 (Table 3). Because the study was cross-sectional, this difference should not be interpreted as evidence that training caused stronger attitudes.

### 3.4 Factors associated with knowledge of local safe-handover arrangements

An exploratory multivariable binary logistic-regression analysis was conducted to examine factors associated with knowledge of where and how unused medicines could be handed over for safe disposal in the participant’s city. The analysis was based on 72 complete cases because two participants had missing predictor data.

The overall model was statistically significant, likelihood-ratio χ^2^(9) = 24.409, *p* = 0.004. The model explained 28.8% of the outcome variance according to the Cox–Snell R^2^ and 38.7% according to the Nagelkerke R^2^. It correctly classified 76.4% of participants. The Hosmer–Lemeshow test did not indicate poor model fit, χ^2^(8) = 3.480, *p* = 0.901.

Year of study was associated with local safe-handover knowledge as an overall predictor (Wald χ^2^(4) = 12.622, *p* = 0.013). Third-year students had higher odds of reporting local safe-handover knowledge than first-year students, adjusted OR = 14.28, 95% CI 2.59–78.58, *p* = 0.002. The comparisons between first-year students and second-year students, adjusted OR = 1.72, 95% CI 0.32–9.39, *p* = 0.529; fourth-year students, adjusted OR = 4.36, 95% CI 0.44–43.18, *p* = 0.208; and fifth-year students, adjusted OR = 7.69, 95% CI 0.79–75.40, *p* = 0.080, were not statistically significant.

Familiarity with sustainable pharmacy was not associated with local safe-handover knowledge as an overall predictor, *p* = 0.274. Compared with participants who were not familiar with sustainable pharmacy, the adjusted odds were not significantly different among partially familiar participants, adjusted OR = 0.92, 95% CI 0.22–3.92, *p* = 0.911, or familiar participants, adjusted OR = 3.42, 95% CI 0.55–21.27, *p* = 0.187.

Awareness of Bulgarian sustainability regulation was not associated with the outcome as an overall predictor, *p* = 0.913. Previous participation in sustainability training was also not independently associated with local safe-handover knowledge, adjusted OR = 1.73, 95% CI 0.27–11.14, *p* = 0.562 (Table 4).

**Table 4:** Multivariable logistic regression of factors associated with knowledge of local safe-handover arrangements for unused medicines.

Predictor	Category comparison	Adjusted OR	95% CI	*p*-value
Familiarity with sustainable pharmacy	Overall effect	—	—	0.274
	Partially familiar vs. not familiar	0.92	0.22–3.92	0.911
	Familiar vs. not familiar	3.42	0.55–21.27	0.187
Awareness of Bulgarian regulation	Overall effect	—	—	0.913
	Do not know vs. no	1.40	0.14–14.19	0.777
	Yes vs. no	1.67	0.15–18.74	0.679
Previous sustainability training	Yes vs. no	1.73	0.27–11.14	0.562
Year of study	Overall effect	—	—	**0.013**
	Second vs. first year	1.72	0.32–9.39	0.529
	Third vs. first year	14.28	2.59–78.58	**0.002**
	Fourth vs. first year	4.36	0.44–43.18	0.208
	Fifth vs. first year	7.69	0.79–75.40	0.080

Bold values indicate statistical significance at *p* < 0.05.

The confidence intervals around several estimates were wide, indicating limited precision. The regression findings should therefore be interpreted as exploratory, particularly given the sample size, the number of categories, and the number of parameters included in the model.

## 4 Discussion

This study examined sustainability-related awareness, educational exposure, attitudes, and knowledge of medication disposal among pharmacy students at three Bulgarian universities. Three principal findings emerged. First, students generally regarded pharmacists’ engagement in sustainability as important, but only 14 (18.9%) reported previous participation in sustainability-related training. Second, familiarity with sustainable pharmacy was associated with both previous training and year of study. Third, knowledge of medicine-disposal arrangements varied depending on how it was measured: 32 students (43.2%) knew where and how to safely hand over unused medicines in their city, whereas 43 (58.1%) reported broader knowledge of where to dispose of unwanted, expired, or damaged medicinal products.

These findings should be interpreted in light of a broad understanding of pharmaceutical sustainability. Sustainable pharmacy encompasses not only the disposal of unused medicines but also environmentally responsible pharmaceutical development and manufacturing, procurement, supply, prescribing, dispensing, medicine use, and end-of-life management. It further includes rational use of medicines, prevention of avoidable pharmaceutical consumption, resource stewardship, and consideration of environmental effects in professional decision-making (15). The present questionnaire assessed selected aspects of this broader construct, particularly awareness, education, attitudes, and medicine disposal. It did not measure the full range of sustainability competencies. Consequently, the findings should not be interpreted as a comprehensive assessment of students’ pharmaceutical-sustainability competence.

The combination of positive attitudes and limited educational exposure is consistent with international evidence. In Australia, Chen et al. found that pharmacy students generally considered environmental sustainability relevant to professional practice and recognized a responsibility for the profession to act, despite limited knowledge and curricular exposure (7). Canadian pharmacy students have likewise expressed strong interest in learning about the environmental consequences of medicines while reporting limited curricular coverage (7). More recent qualitative research found that pharmacy students viewed sustainability as professionally important but perceived it as insufficiently integrated into their education and future practice (16). The agreement between these studies and the present findings suggests that positive attitudes may develop before students acquire structured knowledge or practical skills.

A recent systematic review of 15 studies from 11 countries reached a similar conclusion: pharmacy students and professionals generally recognized environmental risks, but knowledge gaps persisted in medicine disposal, ecopharmacovigilance, and broader sustainability concepts. Common barriers included insufficient formal education, unclear guidance, and limited institutional support (17). The Bulgarian findings therefore appear to reflect a broader international gap between recognition of sustainability as a professional responsibility and preparedness to apply it in practice.

Nevertheless, comparisons between studies require caution. Sustainability has been operationalized differently across surveys. Some studies examine climate change, pharmaceutical pollution, or ecopharmacovigilance, whereas others assess waste management, sustainable prescribing, or general environmental attitudes. The present study used individual self-reported questions rather than a validated multidimensional competency instrument. Differences between countries may also reflect variation in curricula, medicine take-back systems, local disposal infrastructure, professional standards, and public awareness campaigns rather than differences in student motivation alone.

The association between previous training and familiarity with sustainable pharmacy was relatively strong, Cramér’s V = 0.532. Students reporting training also had higher attitude scores than untrained students, although the effect size was small to moderate, r = 0.25. These results are compatible with the possibility that formal or extracurricular educational exposure supports awareness and engagement. Educational studies provide some support for this interpretation. Gruenberg et al. (18) demonstrated the feasibility of incorporating pharmaceutical sustainability into a required pharmacy course through preparatory activities, an interactive lecture, professional discussion, and case-based learning. However, the cross-sectional design of the present study does not establish that training led to greater familiarity or stronger attitudes. Students already interested in sustainability may have been more likely to seek relevant courses or seminars.

The relationship between study year and sustainability outcomes was more complex than a simple pattern of curricular progression would suggest. Fourth- and fifth-year students more frequently reported familiarity, but the adjusted regression did not demonstrate a monotonic increase in local safe-handover knowledge across study years. Only the comparison between third- and first-year students was statistically significant, and its confidence interval was wide, adjusted OR = 14.28, 95% CI 2.59–78.58. Second-, fourth-, and fifth-year comparisons were not statistically significant. These results do not support the conclusion that sustainability competence develops progressively through the curriculum. They may reflect differences between student cohorts, informal learning, practice exposure, extracurricular activities, or statistical instability associated with the small groups.

Previous training was not independently associated with local safe-handover knowledge in the multivariable model, adjusted OR = 1.73, 95% CI 0.27–11.14. This does not necessarily contradict its association with familiarity and attitudes. The analyses assessed different outcomes: knowing the term, valuing pharmacists’ engagement, and knowing local disposal arrangements are related but distinct constructs. A course or seminar may increase conceptual awareness without providing information about locally available collection points. Conversely, students may learn disposal arrangements through employment, community experience, public information, or personal medicine use. The wide confidence interval also indicates that the study had limited precision to detect an independent association. Similar knowledge–practice gaps have been observed in Kosovo, where pharmacy and nursing students recognized the environmental risks of improper disposal but frequently discarded medicines in household waste and rarely returned them to pharmacies (19).

The distinction between the two disposal questions is particularly important. Fewer than half of participants knew where and how medicines could be handed over safely in their city, while a larger proportion reported general knowledge of where unwanted or expired medicines should be discarded. The difference may indicate that students possess general knowledge but lack confidence about locally available arrangements. It may also result from differences in question wording. Future studies should distinguish knowledge of recommended disposal principles from knowledge of accessible local collection infrastructure and should verify knowledge using objective questions where possible. Unsafe disposal has also been reported among pharmacy and nursing students in Saudi Arabia, where most respondents discarded expired medicines in household waste or flushed them into sinks or toilets (20).

The distinction between knowledge and behavior is important. Al Rawwad et al. (21) found that although many pharmacy and nursing students knew the recommended disposal methods, their personal disposal practices did not consistently reflect that knowledge. A Nigerian study similarly found that knowledge did not necessarily translate into safe practice. Previous training was associated with better disposal practices, while limited access to medicine take-back programs was the most frequently reported barrier (21).

Limited sustainability training is not restricted to undergraduate students. In a survey of 440 pharmacy professionals and trainees from 24 countries, most respondents reported no previous climate-related training, while lack of knowledge, time, and resources were important barriers to action (22).

The Bulgarian context gives these findings practical relevance, but it also limits the conclusions that can be drawn. Students were recruited from three universities, providing broader institutional representation than a single-site study, but the convenience sample cannot be considered nationally representative. Furthermore, this survey did not audit course syllabi, learning outcomes, teaching hours, or assessment methods. It therefore cannot establish that sustainability is absent from Bulgarian pharmacy curricula. A national curriculum-mapping study would be needed to determine where sustainability topics are taught and whether coverage includes the complete pharmaceutical life cycle.

International curriculum studies offer useful models for such work. Canadian educators reported that only a minority of respondents included environmental sustainability content, and that it was commonly delivered through lectures (9, 10). In Sweden, curriculum reviews and educator surveys found that environmental topics were included in some pharmacy courses but were unevenly integrated across programs (12, 13). Applying a comparable curriculum-mapping approach in Bulgaria could identify existing content, duplication, gaps, and opportunities for longitudinal integration.

The findings support consideration of structured sustainability learning outcomes within Bulgarian pharmacy education. Relevant content could include pharmaceutical pollution, sustainable manufacturing and procurement, rational and environmentally informed medicine use, medicine take-back systems, patient counseling, waste prevention, environmental risk communication, and the pharmacist’s role in public health (23). Teaching strategies could combine foundational instruction with case-based learning, simulated patient counseling, medicine-waste audits, and experiential activities. Interactive and practice-oriented approaches may be particularly valuable. A Dutch educational module integrating e-learning and patient cases into a pharmacy simulation significantly increased students’ knowledge of pharmaceutical residues in water (24). These recommendations should be regarded as educational implications requiring evaluation rather than interventions whose effectiveness was demonstrated by the present study.

This study has several limitations. Its cross-sectional design prevents causal inference, and students from different years represent different cohorts rather than longitudinal stages of the same group. Convenience sampling and voluntary participation may have produced selection bias, particularly if students interested in sustainability were more likely to respond. The sample was small, some categories contained few observations, and the regression model included several parameters based on 72 complete cases. The resulting wide confidence intervals indicate limited precision and possible model instability. The questionnaire used self-reported, primarily single-item measures and did not undergo documented comprehensive psychometric validation. The study also assessed selected aspects of sustainability rather than objective or multidimensional competence. Future research should use validated instruments, objective knowledge measures, curriculum mapping, larger multicenter samples, and longitudinal or educational-intervention designs.

## 5 Conclusion

This study demonstrates that future pharmacists recognize the importance of sustainability in pharmaceutical practice, yet their educational exposure and practical knowledge remain limited.

While students expressed strong support for pharmacists’ engagement in sustainable activities, gaps in formal training indicate that sustainability is not systematically embedded within pharmacy education. Educational experience was associated with improved awareness, attitudes, and knowledge, highlighting education as a key driver of pharmaceutical sustainability. Integrating structured sustainability content, including medicine waste management, environmental stewardship, and sustainable decision-making, into pharmacy curricula is essential to prepare a workforce capable of supporting environmentally responsible healthcare systems. Strengthening sustainability education may enhance professional competence, promote responsible use and disposal of medicine, and contribute to broader public health and environmental goals.

## Data Availability

The original contributions presented in the study are included in the article/supplementary material, further inquiries can be directed to the corresponding author.
